# Multicenter observational survey on psychosocial and behavioral impacts of COVID-19 in people living with HIV in Northern Vietnam

**DOI:** 10.1038/s41598-023-47577-9

**Published:** 2023-11-21

**Authors:** Shoko Matsumoto, Moeko Nagai, Linh Khanh Tran, Kazue Yamaoka, Hoai Dung Thi Nguyen, Trang Dinh Van, Junko Tanuma, Thach Ngoc Pham, Shinichi Oka, Giang Van Tran

**Affiliations:** 1 AIDS Clinical Center, National Center for Global Health and Medicine, Tokyo, Japan; 2 Graduate School of Public Health, Teikyo University, Tokyo, Japan; 3 National Hospital for Tropical Diseases, Hanoi, Vietnam; 4 Hanoi Medical University, Hanoi, Vietnam

**Keywords:** HIV infections, Epidemiology, Quality of life

## Abstract

Socially marginalized groups, including people living with HIV/AIDS (PLHIV), could be disproportionately affected by Coronavirus disease 2019 (COVID-19). Following an initial single-center survey conducted in 2020, we conducted a second survey of 11 antiretroviral therapy (ART) sites in Northern Vietnam between June 2021 and January 2022. We tested anti-SARS-CoV-2 (severe acute respiratory syndrome coronavirus 2) nucleocapsid IgG antibodies and assessed prevention against COVID-19 and impacts of COVID-19 on access to ART, economic security, risky health behaviors, and mental health using self-reported questionnaires. In total, 7808 PLHIV on ART participated in the second survey. The overall prevalence of SARS-CoV-2 antibody was as low as 1.2%. There was no clear upward trend in COVID-19 infection among PLHIV compared with the rate of infection among the general population. HIV treatment was generally maintained and no increase in risky health behaviors was observed. The economic impacts were significant, with high unemployment rate, poorer economic security, and binge drinking strongly associated with depression. However, the prevalence of depression decreased by 11.2% compared with pre-COVID-19 levels. Social support, including for patients to continue HIV treatment and effective employment/financial assistance, may help to alleviate the negative socioeconomic impacts of COVID-19 and improve mental health among PLHIV.

## Introduction

The pandemic of coronavirus disease 2019 (COVID-19) has been ongoing since early 2020 and the effects have been far-reaching, affecting not only individual health but also economies and health care systems^1–3^. Socially marginalized groups according to race and ethnicity, sex, income, and geographic region often bear a disproportionately large burden^4–7^. People living with HIV/AIDS (PLHIV), who are often drug users, sexual minorities, and sex workers, face numerous socioeconomic challenges (e.g., poverty, limited income opportunities, stigma, and discrimination)^8,9^. PLHIV could be affected by COVID-19 in many ways, including elevated risk of severe COVID-19 outcomes and mortality^10,11^, interruption of HIV treatment and care^12,13^, and increased HIV risk behavior^14^.

In the early phase of the COVID-19 pandemic (June–July 2020), we conducted a cross-sectional study of anti-SARS-CoV-2 (severe acute respiratory syndrome coronavirus 2) nucleocapsid (N) IgG antibodies and psychosocial and behavioral impacts of COVID-19 in a large HIV outpatient clinic at the National Hospital for Tropical Diseases (NHTD) in Hanoi, Vietnam. In that study of 1243 PLHIV, we found that the impacts of COVID-19 were small with respect to access to antiretroviral therapy (ART) but were substantial in terms of economic security and risky health behaviors, which were associated with psychological stress^15^.

Beginning in April 2021, Vietnam experienced its largest COVID-19 outbreak, the fourth epidemic wave in the country, which has resulted in over 11 million infections as of March 2023^16^. In response, many regions in Vietnam enforced strict citywide lockdowns and stay-at-home orders until the country entered a more normal stage in October 2021^17^. After restrictions were eased, Vietnam recorded its first case of infection involving the SARS-CoV-2 Omicron variant in December 2021, leading to a peak of infection, with the highest number of new cases (454,212 cases) recorded on 12 March 2022^16^.

We scaled up our previous survey and conducted a second survey at 11 ART sites in Northern Vietnam during the period with strict social restrictions in the fourth COVID-19 outbreak during 2021. In the second survey, we aimed to explore the long-term impacts of COVID-19 and changes in the impacts of different pandemic phases. In particular, we assessed anti-SARS-CoV-2 N IgG antibodies and psychosocial and behavioral impacts of COVID-19 using a revised questionnaire. Our previous survey reported a prevalence of anti-SARS-CoV-2 N IgG antibodies of 0.2%, but this rate may have been underestimated partly owing to privacy violation concerns in SARS-CoV-2 testing. Additionally, the first survey showed that the economic impact of COVID-19 was enormous and associated with psychological distress and that improved social support may alleviate such psychological impact. However, the need for PLHIV to improve economic security and social support was not specifically evaluated in that survey. To address this limitation in our previous survey, the present survey included questions on barriers to COVID-19 testing, social support needs to continue ART and HIV care during the pandemic, perceived financial status, and preferred forms of assistance. The information from this study could help with understanding how PLHIV are affected by the COVID-19 pandemic and developing appropriate strategies to meet their needs.

## Methods

### Study design and study participants

From June 2021 to January 2022, we conducted SARS-CoV-2 antibody testing and administered a self-report questionnaire survey on the psychosocial and behavioral impacts of COVID-19 among PLHIV (aged ≥ 18 years) at 11 ART facilities in Northern Vietnam. These facilities were involved in an HIV research project entitled, “Establishment of the “bench-to-bedside” feedback system for sustainable ART and the prevention of new HIV transmission in Vietnam,” which started in 2019 under a Japanese government program, the “Science and Technology Research Partnership for Sustainable Development” (SATREPS). These 11 ART facilities were selected in consultation with the Vietnamese Ministry of Health from multiple perspectives, including region, facility level, HIV prevalence, and support from other overseas donors. Additionally, owing to SATREPS being implemented within the framework of the Japanese government’s official development assistance, some facilities were selected with an intention to provide technical assistance to facilities with insufficient access to HIV services, such as viral load testing. The study sites include one national-level hospital (NHTD), seven provincial/city-level hospitals, and three district-level hospitals. Four of the 11 hospitals were located in Hanoi (Table 1).

**Table 1:** Study participation and incidence of SARS-CoV-2 infection by study site.

	Study participants	Participation rate^a^	Anti-SARS-CoV-2 nucleocapsid antibodies		
			Test number	Sample collection period	Positive
	n (%)	%	n	Month year	n (%)
All	7808 (100.0)	90.4	7780	Jun 2021–Jan 2022	94 (1.2)
Hospital (location)					
National level	1116 (14.3)	85.7	1106		9 (0.8)
NHTD (Hanoi)	1116 (14.3)	85.7	1106	Jun 2021–Jan 2022	9 (0.8)
Provincial/city level	4937 (63.2)	89.6	4919		60 (1.2)
QNGH (Quang Ninh)	1189 (15.2)	97.9	1188	Sep–Nov 2021	17 (1.4)
HDHTD (Hai Duong)	927 (11.9)	82.1	911	Jun–Aug 2021	2 (0.2)
DDGH (Hanoi)	809 (10.4)	92.1	809	Sep 2021–Jan 2022	7 (0.9)
NAGH (Nghe An)	658 (8.4)	85.2	658	Oct–Dec 2021	14 (2.1)
HYTD (Hung Yen)	600 (7.7)	95.2	600	Sep–Dec 2021	4 (0.7)
09 hospital (Hanoi)	406 (5.2)	85.8	406	Sep–Dec 2021	8 (2.0)
HTCDC (Ha Tinh)	348 (4.5)	83.9	347	Oct 2021–Jan 2022	8 (2.3)
District level	1755 (22.5)	96.5	1755		25 (1.4)
NTL (Hanoi)	1515 (19.4)	98.4	1515	Sep–Dec 2021	18 (1.2)
TSMC (Phu Tho)	138 (1.8)	89.0	138	Oct 2021	2 (1.5)
YBMC (Yen Bai)	102 (1.3)	81.6	102	Oct–Nov 2021	5 (4.9)

^a^Participation rate was calculated as the number of participants divided by the total number of outpatients under follow-up at each hospital.

Of all participants, only four (0.05%) and 42 people (0.5%) self-reported that they had ever been diagnosed with SARS-CoV-2 infection in a polymerase chain reaction (PCR) test or had been quarantined previously.

*NHTD* National Hospital for Tropical Diseases, *QNGH* Quang Ninh General Hospital, *HDHTD* Hai Duong Hospital for Tropical Diseases, *DDGH* Dong Da General Hospital, *NAGH* Nghe An General Hospital, *HYTD* Hung Yen Hospital of Tropical Diseases, *09 hospital* 09 hospital, *HTCDC* Ha Tinh Center for Disease Control and Prevention, *NTL* Nam Tu Liem Health Center, *TSMC* Thanh Son District Medical Center, *YBMC* Yen Binh District Medical Center.

The content validity of the revised questionnaire was determined by an expert panel comprising HIV/AIDS experts, including HIV clinicians and social epidemiologists. The panel reviewed each item to ensure that the questionnaire was comprehensive and that no items were inappropriate in the Vietnamese cultural context.

All outpatient PLHIV who visited the study sites during the survey period were invited to complete the survey and to have a blood sample collected for antibody testing during their regular consultations. Individuals who provided written informed consent participated in the survey on the same day as their consultation. Because data collection was carried out during the largest COVID-19 outbreak in Vietnam, those who could not visit the study sites during the study period because of movement restrictions and those who received ART via post or in other facilities could not participate in the survey.

### Measurement

#### Characteristics of study participants

Information on sex, age, and occupation before the COVID-19 pandemic were collected. Age was divided into four categories using interquartile values. Occupation before the pandemic fell into the following categories: salaried worker, self-employed, household worker, unemployed or student or retired, or other.

#### Incidence of SARS-CoV-2 infection

The incidence of SARS-CoV-2 infection was investigated using the questionnaire survey and laboratory-based immunoassay to detect anti-SARS-CoV-2 N IgG antibodies with an automated immunoassay system (ARCHITECT i2000, Abbott Laboratories Inc., Abbott Park, IL, USA) and a 6R86 SARS-CoV-2 IgG Reagent Kit (Abbott Laboratories Inc.). In the questionnaire, the self-reported incidence was assessed by asking whether the respondent had been diagnosed with SARS-CoV-2 infection in a polymerase chain reaction (PCR) test or quarantined as a close contact of another person with such a diagnosis. The methods used for IgG antibody testing were the same as those in a previous study conducted in 2020 at the NHTD and are described elsewhere^15^.

The SARS-CoV-2 IgG assay is designed to detect IgG antibodies to the nucleocapsid (N) protein of SARS-CoV-2 whereas the main target of SARS-CoV-2 vaccines available in Vietnam is the spike (S) protein at the time of the survey. No study participant had received an inactivated whole-cell vaccine containing the N protein. Therefore, our antibody test results were not affected by vaccine history.

#### Prevention against COVID-19 infection

The questionnaire included the total vaccine doses received so far, behaviors to prevent COVID-19 infection (i.e., wearing a mask, hand washing, gargling, social distancing, and others), changes in social contacts compared with those pre-COVID-19 (i.e., no change, reduced, or increased), and willingness to have a diagnostic test when having symptoms of COVID-19 (i.e., willing to be tested, not willing to be tested, or unsure). Participants who said they were not willing to be tested were asked the reasons. The response options included “fear of HIV status being disclosed,” “fear of laboratory confirmation of COVID-19 infection,” and “fear of discrimination against people with COVID-19 infection,” and participants could select all applicable options.

#### ART continuity

The questionnaire assessed participants’ experience of continuing ART during the outbreak (i.e., continued to receive ART without interruption or discontinuation owing to the pandemic) and their experience of receiving ART at another clinic that was not their regular hospital during the outbreak. Question responses were dichotomous. Additionally, participants were asked whether they had received any form of social support to continue ART and HIV care during the COVID-19 pandemic. Participants used free text to describe the type of social support that could be effective in ensuring continuity of ART during the pandemic. For the NHTD, all patients had scheduled visits to measure HIV viral load during June–July each year. We obtained visit status and reasons for missing a scheduled visit, as well as data on HIV viral load in June–July 2020 and 2021.

#### Risky health behaviors

Alcohol intake, drug use, and sexual behaviors were assessed. Regarding alcohol intake, participants were asked whether they had any episodes of binge drinking, defined as > 5 drinks on one occasion^18^, in the past month and any change in alcohol consumption before and after the COVID-19 pandemic. As for drug use, participants were asked whether they had used illegal drugs in the past 3 months and whether there was a change in the amount of drug use before and after the pandemic. Finally, we queried changes in the number of sex partners and frequency of using a condom during sex, before and after the outbreak. For questions asking about these changes, possible responses were “no change,” “decreased,” and “increased.” For the items on alcohol intake and drug use, “non-drinker” and “non-drug user” were added to the response categories.

#### Economic security

Changes in employment status were assessed using five categories (i.e., no change, lost job, reduced working hours, increased working hours, and other). Participants’ pre- and post-COVID-19 household income and their current financial status were also assessed. Financial status was rated according to categories (i.e., no problems, a little challenging, very challenging, and other). We also asked whether participants had ever received financial assistance from any public authority. Finally, participants reported any forms of emergency assistance that could be helpful to support their lives using six options (i.e., no need, cash benefit, food benefit, tax exemption, rent subsidy, and other); participants could select all applicable options.

#### Mental health

In the first survey conducted at the NHTD during 2020, the Vietnamese version of the Depression, Anxiety and Stress Scale-21 (DASS-21-V) with a cutoff score of 34 was used to measure the general distress experienced by study participants^15^. However, one limitation of our previous study was that there was no comparable prevalence of general distress using that cutoff score in the Vietnamese PLHIV population before and after the COVID-19 pandemic.

Prior to the COVID-19 pandemic, the National Center for Global Health and Medicine began a collaboration with NHTD, a study site in the present research, to monitor clinical outcomes of PLHIV. Under this collaboration, we conducted a study to assess depression using the Center for Epidemiological Studies-Depression (CES-D) scale in an HIV patient cohort at the NHTD in 2016^19^. To address limitations of the first survey and compare the prevalence of depression before and after the COVID-19 pandemic, we assessed depression using the CES-D, as well as general distress using the DASS-21-V, among participants in the second survey at the NHTD. The CES-D is a widely used self-reporting scale and there is strong evidence for both its reliability and validity in Vietnam’s HIV population, with a Cronbach’s alpha of 0.81 and sensitivity and specificity of 79.8% and 83.0%, respectively, with a cutoff score of 16^20^. The CES-D comprises 20 items answered on a four-point scale ranging from 0 (rarely or none of the time) to 3 (most or almost all the time), except for four items that are positively worded and scored in reverse. Regardless of whether questions were scored in reverse, if a respondent provided the same answer to all 20 items, the CES-D score was considered invalid and was excluded from the analysis. We used the Vietnamese version of the CES-D, which was previously available^20^. We defined a CES-D score of ≥ 16 as experiencing depression because this cutoff has been proven optimal for assessing depression in Vietnam’s HIV population^20^. The DASS-21-V comprises 21 items answered on a four-point scale ranging from 0 (does not apply to me at all) to 3 (applies to me very much or most of the time). According to the scoring instructions^21^, the total score is calculated by multiplying the sum of the responses by two. In line with the first survey, a cutoff score of 34 was used to measure general distress. This cutoff score was suggested by Tran et al. to detect general distress, including depression and anxiety, with a sensitivity of 79.1% and specificity of 77.0% in Vietnamese women^22^.

### Statistical analysis

First, we descriptively evaluated the incidence of SARS-CoV-2 infection, prevention against COVID-19 infection, impact of COVID-19 on ART continuity, economic security, and risky health behaviors among all study participants. Next, to assess changes in the impacts of COVID-19 during different pandemic phases, we compared differences in responses between the two surveys conducted at the NHTD in 2020 and 2021 using the McNemar test. Additionally, a study on depression among PLHIV patients was previously conducted at the NHTD in 2016, prior to the COVID-19 pandemic. Using that available data, the prevalence of and factors associated with depression were compared between the 2016 survey (pre-COVID-19) and the present 2021 survey (post-COVID-19). Comparisons between the 2020 and 2021 surveys and the 2016 and 2021 surveys were conducted for the same questions or using the same scales among those who participated in both surveys (i.e., the same population). Univariate logistic regression models were used to investigate factors associated with depression, and crude odds ratios (ORs) were calculated. As supplementary analysis, we performed logistic regression models using all data, including from participants who gave the same response to all items on the CES-D.

All analyses were performed using SAS 9.4 software (SAS Institute Inc., Cary, NC, USA). All tests were two-sided, with the significance level set at 5%. Missing data were excluded from the analyses.

### Ethics statement

The study was approved by the Human Research Ethics Committee of the National Center for Global Health and Medicine (reference: NCGM-G-003560-02) and the Bio-medical Research Ethics Committees of the National Hospital for Tropical Diseases (reference: 12/HDDD-NDTU). We performed this study in accordance with Japan’s Ethical Guidelines for Medical and Health Research Involving Human Subjects, issued by the Japanese Ministry of Health, Labour and Welfare.

## Results

### Participation rate and incidence of SARS-CoV-2 infection

Among 8633 HIV-infected outpatients under follow-up at 11 study sites, 7808 participated in the survey, with more than an 80% participation rate in each site and 90.4% as a whole (Table 1). Of 7808 study participants, only four (0.05%) participants self-reported previous SARS-CoV-2 infection confirmed by PCR in the questionnaire survey. However, of 7780 participants who were successfully tested for anti-SARS-CoV-2 N IgG antibodies, 94 (1.2%) were found to be positive. The antibody positivity rates ranged from 0.2% in Hai Duong Hospital for Tropical Diseases in Hai Duong Province to 4.9% in Yen Binh District Medical Center in Yen Bai Province (Table 1).

### Demographics of study participants

In total, 64.6% of study participants were men, with mean age 41.1 years. Fewer than half (46.3%) reported that they were salaried workers before the COVID-19 pandemic; 26.1% and 14.2% reported that they were self-employed and household workers, respectively (Table 2).

**Table 2:** Characteristics of study participants and COVID-19 prevention measures.

Variables	n (%)
Demographics	
Sex	
Male	5044 (64.6)
Female	2764 (35.4)
Age, y	
Mean ± standard deviation	41.1 ± 9.37
< 37	2001 (25.6)
37–41	1994 (25.5)
42–45	1701 (21.8)
≥ 46	2099 (26.9)
N/A	13 (0.2)
Occupation before COVID-19	
Salaried worker	3612 (46.3)
Self-employed	2040 (26.1)
Household worker	1105 (14.2)
Unemployed, student, retired	917 (11.7)
Other^a^	88 (1.1)
N/A	
Prevention against COVID-19	
Vaccine history	
None	2476 (31.7)
One dose	2402 (30.8)
Two doses or more	1739 (22.3)
N/A^b^	1191 (15.3)
COVID-19 prevention measures	
Practiced	7747 (99.2)
Wearing a mask^c^	7590 (97.2)
Hand washing or gargling^c^	7339 (94.0)
Social distancing^c^	7221 (92.5)
Other^c^	197 (2.5)
Change in social contact^d^	
No change	2118 (27.1)
Reduced	5579 (71.5)
Increased	80 (1.0)
N/A	31 (0.4)
Willing to undergo diagnostic test with COVID-19 symptoms	
Yes	7435 (95.2)
No	280 (3.6)
Not sure	57 (0.7)
N/A	36 (0.5)
Reasons for being unwilling to undergo testing^e^	
Fear of HIV status being disclosed	147 (52.5)
Fear of laboratory confirmation of COVID-19 infection	115 (41.1)
Fear of hospitalization or isolation	85 (30.4)
Fear of negative impact of COVID-19 infection on daily life	83 (29.6)
Testing is burdensome	65 (23.2)
Fear of discrimination against people with COVID-19 infection	39 (13.9)
Other	5 (1.8)

^a^Includes employers (n = 71) and union workers (n = 17).

^b^Data were missing owing to accidental failure in data collection in Hai Duong Hospital for Tropical Diseases (n = 923) and National Hospital for Tropical Diseases (n = 267).

^c^Among those who practiced protective behaviors (multiple choices possible).

^d^Compared with before the COVID-19 pandemic.

^e^Among those who said they were unwilling to undergo COVID-19 testing (n = 280).

### Behaviors to prevent COVID-19

Vaccines for SARS-CoV-2 became widely available in Vietnam during the third quarter of 2021^23^. At the time of the survey, more than half of study participants had been vaccinated against SARS-CoV-2 infection (Table 2). The vaccination rate (those who received at least one dose of vaccine) varied among hospitals, ranging from 17.4% in Thanh Son District Medical Center in Phu Tho Province to 88.2% in Ha Tinh Center for Disease Control and Prevention in Ha Tinh Province. This reflected regional differences in vaccine availability. Until a sufficient supply of vaccines was available in Vietnam, provinces with the greatest need for vaccines are prioritized for vaccine procurement, taking into consideration the number of infections and areas at risk of outbreak.

Most participants (99.2%) were practicing preventive measures, including wearing a mask (97.2%) and hand washing or gargling (94.0%). Additionally, 71.5% reported having less social contact than before the COVID-19 pandemic. Furthermore, most participants (95.2%) reported being willing to undergo diagnostic testing for SARS-CoV-2 infection when symptoms appeared; 280 (3.6%) were unwilling to be tested. The most frequently reported reasons for reluctance to undergo testing for COVID-19 were fear of HIV status being disclosed (52.5%) and fear of laboratory confirmation of COVID-19 infection (41.1%). Few participants were reluctant to be tested for fear of discrimination against those infected with SARS-CoV-2 (Table 2).

### ART continuity

Most study participants (99.1%) continued ART without interruption during the COVID-19 pandemic. Only a few participants (1.4%) reported that they temporarily received ART at another hospital during the COVID-19 outbreak. Most participants (73.2%) reported receiving social support to continue ART during the COVID-19 pandemic; the remaining participants (26.8%) reported receiving no support (Table 3). The proportion of patients receiving such support varied among study sites. More than 90% of study participants in seven study sites reported receiving support; however, the proportion was less than half in the other four sites (Supplementary Table S1). Additionally, 3484 participants provided comments regarding their needs with respect to effective social support to continue HIV treatment during the COVID-19 pandemic. The most frequent comments were related to continuous provision of ART and HIV services (61.9%) and special arrangements to continue ART (11.5%), which included multi-month prescriptions and drug delivery via post or shipping. These were followed by provision of informational support related to COVID-19 and HIV services (11.3%), and financial and material support (10.4%) including provision of cash, material, free testing, and transportation (Supplementary Table S2).

**Table 3:** Socio-behavioral impacts of COVID-19 during the fourth outbreak in 2021.

Variables	n (%)
ART continuity	
Continuation of ART during COVID-19	
Continued without interruption	7734 (99.1)
Discontinued owing to COVID-19	56 (0.7)
N/A	18 (0.2)
Access to ART during COVID-19	
Continued receiving ART from patient’s regular hospital	7677 (98.3)
Temporarily received ART at another hospital	109 (1.4)
N/A	22 (0.3)
Support for continuing ART and HIV care during COVID-19	
Received	5718 (73.2)
Not received	2090 (26.8)
Risky health behaviors	
Binge drinking in the past month	
> 5 drinks on one occasion	1078 (13.8)
No episodes of having > 5 drinks on one occasion	6720 (86.1)
N/A	10 (0.1)
Change in alcohol consumption^a^	
Non-drinker	5903 (75.6)
No change	566 (7.3)
Decreased	1272 (16.3)
Increased	58 (0.7)
N/A	9 (0.1)
Used illegal drugs in the past 3 months	
Non-drug user	7548 (96.7)
Used	253 (3.2)
N/A	7 (0.1)
Change in illicit drug use after COVID-19^a^	
Non-drug user	7485 (95.9)
No change	86 (1.1)
Decreased	216 (2.8)
Increased	6 (0.1)
N/A	15 (0.2)
Change in the number of sex partners after COVID-19^a^	
No change	7321 (93.8)
Decreased	364 (4.7)
Increased	58 (0.7)
N/A	65 (0.8)
Change in condom use after COVID-19^a^	
No change	7146 (91.2)
Increased	333 (4.3)
Decreased	136 (1.7)
N/A	193 (2.5)
Economic impact	
Change in employment status after COVID-19^a^	
No change	4134 (53.0)
Lost job	1393 (17.8)
Reduced working hours	2020 (25.9)
Increased working hours	55 (0.7)
Other^b^	150 (1.9)
N/A	56 (0.7)
Pre-COVID household income (VND^c^)	
Median (IQR)	6,000,000 (4,200,000, 10,000,000)
Post-COVID household income (VND)	
Median (IQR)	4,000,000 (1,800,000, 7,000,000)
Current financial status	
No problem	2657 (34.0)
A little challenging	3373 (43.2)
Very challenging	1760 (22.5)
Other	4 (0.1)
N/A	14 (0.2)
Financial assistance from public authorities	
Received	391 (5.0)
Never received	7391 (94.7)
N/A	26 (0.3)
Appropriate forms of emergency assistance (multiple choice)	
No need	3161 (40.5)
Cash benefit	4050 (51.9)
Food benefit	248 (3.2)
Tax exemption	121 (1.6)
Rent subsidy	122 (1.6)
Other	56 (0.7)
N/A	50 (0.6)

^a^Compared with before the COVID-19 pandemic.

^b^Including those who newly started working (n = 28), changed jobs (n = 69), and other (n = 53).

^c^Vietnamese dong (VND). 1 USD is equivalent to approximately 23,000 VND.

*ART* antiretroviral therapy, *IQR* interquartile range.

### Risky health behaviors

A total of 1078 participants (13.8%) reported binge drinking in the previous month, defined as having 5 or more drinks on one occasion. Additionally, 253 (3.2%) reported illicit drug use in the past 3 months. However, few participants reported an increase in alcohol consumption (0.7%) or illicit drug use (0.1%) after the COVID-19 pandemic; instead, more participants reported a reduction in the amount of alcohol consumption (16.3%) and illicit drug use (2.8%). Furthermore, fewer participants reported an increased number of sexual partners (0.7%) or decreased condom use when having sex (1.7%) than those who reported opposite changes (4.7% and 4.3%, respectively) (Table 3).

### Economic security

Nearly half of study participants experienced a change in employment status after the COVID-19 outbreak. Among those, 2020 (25.9%) reported that they had reduced working hours and 1393 (17.8%) lost their job. Median household income after COVID-19 decreased by 2 million VND (33.3%) compared with pre-COVID-19 levels. Nearly two-thirds of participants reported that their financial situation was “a little challenging” (43.2%) or “very challenging” (22.5%). However, only 391 (5.0%) had ever received public financial assistance. More than half of participants (51.9%) reported cash benefits as being the most appropriate form of emergency assistance. Notably, participants from the study site in Hanoi reported higher unemployment due to COVID-19 compared with participants from other provinces (26.9% vs. 9.5%). However, there was no difference in perceived financial difficulties and likelihood of receiving financial support from public authorities (Supplementary Table S3).

### Comparison between first and second surveys in the NHTD

In the NHTD, 1110 participants completed both surveys conducted in 2020 and 2021. All these participants had been registered in a hospital-based cohort under SATREPS and their clinical data including HIV viral load were collected every 6 months (two visit cycles, June–July and December–January). Table 4 shows a comparison of results between the two surveys. In general, the tendency of responses did not change between surveys. However, the incidence of SARS-CoV-2 increased slightly, and more participants reported decreased social contact in 2021.

**Table 4:** Comparison of survey results between 2020 and 2021 at the NHTD.

	2020 survey^a^ (n = 1110)	2021 survey^a^ (n = 1110)	*p*^b^
Incidence and prevention of SARS-CoV-2 infection			
History of SARS-CoV-2 infection (self-reported)			
Never diagnosed	1102 (100.0)	1102 (100.0)	–
Diagnosed via PCR	0 (0.0)	0 (0.0)	
Quarantined	0 (0.0)	0 (0.0)	
Anti-SARS-CoV-2 nucleocapsid IgG antibody			
Positive	3 (0.3)	9 (0.8)	0.01
Prevention measures against COVID-19			
Practiced	1056 (97.8)	1064 (98.5)	0.19
Change in social contacts^c^			
No change	496 (47.2)	287 (27.3)	< 0.001
Reduced	547 (52.0)	755 (71.8)	
Increased	9 (0.9)	10 (1.0)	
ART continuity			
Missed scheduled visit (Jun/Jul 2020 vs. Jun/Jul 2021)^d^			
No missed scheduled visits	1239 (96.2)	1026 (79.7)	< 0.001
Missed scheduled visit with known reason^e^	31 (2.4)	247 (19.2)	
Missed scheduled visit without known reason^f^	18 (1.4)	15 (1.2)	
Discontinuation of ART because of COVID-19			
Discontinued	9 (0.9)	16 (1.5)	0.13
HIV viral load (copies/mL)			
< 20	994 (89.6)	1018 (91.7)	0.24
20–199	102 (9.2)	87 (7.8)	
200–999	6 (0.5)	1 (0.1)	
≥ 1000	8 (0.7)	4 (0.4)	
Support for continuing ART and HIV care during COVID-19			
Received	858 (82.3)	1022 (98.1)	< 0.001
Risky health behaviors			
Change in alcohol consumption^c^			
Non-drinker or no change	899 (83.5)	854 (79.3)	< 0.01
Decreased	172 (16.0)	219 (20.3)	
Increased	6 (0.6)	4 (0.4)	
Change in illicit drug use^c^			
Non-drug user	1067 (99.4)	1065 (99.2)	0.55
No change	1 (0.1)	1 (0.1)	
Decreased	6 (0.6)	8 (0.7)	
Increased	0 (0.0)	0 (0.0)	

*NHTD* National Hospital for Tropical Diseases, *ART* antiretroviral therapy, *PCR* polymerase chain reaction, *Ig* immunoglobulin.

^a^The 2020 survey was conducted from June to July 2021. The 2021 survey was conducted from June 2021 to January 2022. The 1110 participants who completed both the 2020 (total participants = 1243) and 2021 (total participants = 1116) surveys were included. Responses to questions included in both the 2020 and 2021 surveys were compared.

^b^McNemar test.

^c^Compared with before the COVID-19 pandemic.

^d^All NHTD patients had scheduled visits during June–July each year. Visit status in June–July 2020 was used for the 2020 survey and that in June–July 2021 for the 2021 survey. The rate was calculated by dividing each number by the number of patients under follow-up at the end of 2019 (n = 1288) before the COVID-19 pandemic.

^e^Missed scheduled visit owing to death, transfer, and social distancing policy. There were no deaths owing to SARS-CoV-2 infection.

^f^Medical staff could not contact the patient.

Regarding ART continuity, among 1288 HIV outpatients who were under follow-up at the end of 2019 before the COVID-19 pandemic, the number who missed a scheduled visit for known reasons increased significantly during the 1-year period between June–July 2020 (n = 31, 2.4%) and June–July 2021 (n = 247, 19.2%). In June–July 2021, 129 PLHIV outpatients (10.0%) could not access the NHTD because of movement restrictions under the COVID-19 social distancing policy, and 106 patients (8.2%) were transferred to other hospitals to receive ART. However, there was no significant difference in missed scheduled appointments without a known reason (1.4% in 2020 vs. 1.2% in 2021). ART discontinuation was rarely reported in both surveys (0.9% in 2020 vs. 1.5% in 2021). Among those who were able to access the NHTD and underwent laboratory testing, HIV viral load was also minimally changed (viral suppression rate: 89.6% in 2020 vs. 91.7% in 2021). During the prolonged COVID-19 pandemic, more participants reported receiving social support to continue ART and HIV care in the 2021 survey (82.3 in 2020 vs. 98.1% in 2021). As for risky health behaviors, more participants reported reduced alcohol consumption in the 2021 survey than in the 2020 survey; no significant change in illicit drug use was observed between the surveys. The responses to other questions, such as changes in sexual behaviors (e.g., number of sexual partners and condom use) and changes in economic conditions (e.g., employment) differed between the two surveys. Therefore, the responses could not be directly compared.

### Mental health

Mental health was assessed only among participants in the NHTD. Table 5 shows the changes in general distress among 1110 study participants who completed both surveys in 2020 and 2021, and the changes in depression among 822 who completed both surveys conducted in 2016 and 2021. The prevalence of general distress (DASS-21-V ≥ 34) increased during the fourth epidemic wave (7.5% in 2020 vs, 8.8% in 2021); however, the difference was not statistically significant. In contrast, the prevalence of depression was decreased by more than 10% between surveys (27.9% in 2016 vs. 16.7% in 2021); the rate was lower in the survey conducted after the start of the COVID-19 pandemic.

**Table 5:** Comparison of mental health status between 2016, 2020, and 2021 in the NHTD.

	2016 survey^a^	2020 survey^a^	2021 survey^a^	*p*^b^
	Pre-COVID	Post-COVID		
General distress (DASS-21-V)^c^				
≥ 34	–	66 (7.5)	78 (8.8)	0.24
Depression (CES-D)^d^				
≥ 16	229 (27.9)	–	137 (16.7)	< 0.001

*NHTD* National Hospital for Tropical Diseases.

^a^The 2016 survey was conducted from January to December 2016. The 2020 survey was conducted from June to July 2021. The 2021 survey was conducted from June 2021 to January 2022.

^b^McNemar test.

^c^DASS-21-V: Vietnamese version of the Depression, Anxiety, and Stress Scale-21. The prevalence of general distress was calculated in the 1110 study participants from the NHTD who completed both the 2020 survey (total participants = 1243) and 2021 (total participants = 1116) surveys.

^d^CES-D: Center for Epidemiological Studies-Depression. The prevalence of depression was calculated in the 822 study participants from the NHTD who gave valid responses to both the 2016 (total participants = 1208) and 2021 (total participants = 1116) surveys.

In the logistic regression analyses for depression in the 2021 survey, 119 (10.7%) participants were excluded from the analyses because they provided the same responses to all 20 items on the CES-D. Table 6 shows the results of univariate logistic regression analyses. Binge drinking in the past month (odds ratio [OR] = 2.04, 95% confidence interval [CI]: 1.29–3.23 vs. no binge drinking); lost job, reduced working hours, or increased working hours owing to COVID-19 (OR = 4.14, 95% CI 2.69–6.37; OR = 1.64, 95% CI 1.03–2.63; OR = 4.63, 95% CI 1.32–16.27, respectively, vs. no change in employment status); and perceived current financial status “a little challenging” or “very challenging” (OR = 3.61, 95% CI 2.17–6.01; OR = 7.28, 95% CI 4.18–12.68, respectively, vs. “no problem”) were significantly associated with a higher proportion of having depression. Supplementary logistic regression analyses where we used all data, including those of participants who gave the same response to all items on the CES-D, did not show any change in the associations (data not shown).

**Table 6:** Univariate logistic regression model showing factors associated with depression.

	Univariate model^a^ (n = 997)	
	OR (95% CI)	*p*
Demographics		
Sex (vs. male)		
Female	1.00 (0.72–1.41)	0.98
Age, y (vs. aged < 37 years)		
37–41	1.03 (0.61–1.73)	0.72
42–45	0.82 (0.48–1.40)	
≥ 46	0.85 (0.50–1.42)	
Prevention against COVID-19		
Prevention against COVID-19 (vs. not practiced)		
Practiced	0.58 (0.16–2.16)	0.42
Change in social contacts (vs. no change)^b^		
Reduced	1.22 (0.82–1.82)	0.55
Increased	0.67 (0.08–5.47)	
ART continuity		
Access to ART during COVID-19 (vs. continued receiving ART from patient’s regular doctor)		
Temporarily received ART at another hospital	1.47 (0.81–2.67)	0.21
Discontinuation of ART because of COVID-19 (vs. not discontinued)		
Discontinued	1.75 (0.56–5.50)	0.34
Support for continuing ART and HIV care during COVID-19 (vs. received)		
Not received	0.36 (0.05–2.77)	0.33
Risky health behaviors		
Binge drinking in the past month (vs. no episodes of having > 5 drinks)		
> 5 drinks on one occasion	2.04 (1.29–3.23)	< 0.01
Change in alcohol consumption (vs. non-drinker/no change)^b^		
Decreased	1.43 (0.97–2.12)	0.08
Increased	3.74 (0.62–22.65)	
Used illegal drug in the past 3 months (vs. no use)		
Used	0.64 (0.08–5.16)	0.68
Change in illicit drug use (vs. non-user/no change)^b^		
Decreased	1.02 (0.12–8.78)	0.99
Increased	–	
Change in the number of sex partners (vs. no change)^b^		
Decreased	1.38 (0.29–6.59)	0.92
Increased	1.11 (0.13–9.55)	
Change in condom use (vs. no change)^b^		
Increased	2.68 (0.24–29.77)	0.57
Decreased	1.79 (0.36–8.95)	
Economic impact		
Change in employment status (vs. no change)^b^		
Lost job	4.14 (2.69–6.37)	< 0.001
Reduced working hours	1.64 (1.03–2.63)	
Increased working hours	4.63 (1.32–16.27)	
Other	1.74 (0.69–4.36)	
Current financial status (vs. no problem)		
A little challenging	3.61 (2.17–6.01)	< 0.001
Very challenging	7.28 (4.18–12.68)	
Other	–	
Financial assistance from public authorities (vs. never received)		
Ever received	0.71 (0.33–1.51)	0.37

*CES-D* Center for Epidemiological Studies-Depression, *OR* odds ratio, *CI* confidence interval.

^a^Among 1116 study participants from the NHTD, 119 (10.7%) were excluded from the analyses because they provided the same answers to all 16 items on the CES-D.

^b^Compared with before the COVID-19 pandemic.

## Discussion

After the initial survey in 2020 at a single center, the second survey was expanded to 11 ART sites in Northern Vietnam. This extensive survey was conducted during the period with strict social restrictions during the largest outbreak of COVID-19 in Vietnam. Using a long-standing cohort of PLHIV before and during the COVID-19 pandemic, the second survey evaluated the long-term impacts of COVID-19 and changes in these impacts between different pandemic phases among PLHIV on ART.

HIV treatment was maintained amidst the stringent social restrictions imposed during the fourth COVID-19 epidemic wave. A comparison of two surveys conducted in 2020 and 2021 at the NHTD showed no increase in loss to follow-up for unknown reasons or virological failure. Only 1.2% of patients missed scheduled visits without a known reason, and more than 90% maintained an undetectable HIV viral load. Since the early days of the COVID-19 outbreak, the NHTD has made substantial efforts to follow up with patients via phone and apps, making tailored adjustments for patients to ensure that they continue to receive ART. Such support is believed to have been effective in preventing patients’ disengagement from HIV care. The Vietnam Administration for HIV/AIDS Control of the Ministry of Health issued a variety of decision documents outlining special measures to ensure HIV continuum of care during the COVID-19 pandemic. However, some participants in our study reported that they needed more flexibility in terms of delivery and pick up of medicine and multi-month prescriptions. Personalized support to meet patients’ needs and innovative strategies to strengthen ART delivery systems^24^ would facilitate sustainable access to ART during an emergency.

The 2021 survey revealed a SARS-CoV-2 antibody positivity rate of 1.2%. This prevalence was lower than that found in previous studies of PLHIV conducted in other countries over a similar period (3.7% in the United States^25^, 27.7% in Guinea-Bissau^26^, 30.6% in Kenya^27^, and 54.6% in India^28^). To our knowledge, ours is the only study to investigate the seroprevalence of SARS-CoV-2 in PLHIV in Vietnam. It is evident that the seroprevalence of SARS-CoV-2 varies greatly between countries and regions within a country, depending on the prevalent virus strain, outbreak phases, and timing of survey. The fact that this study was conducted before emergence of the SARS-CoV-2 Omicron variant in Vietnam, and that the strong social restriction measures were continued by the Vietnamese government, may have contributed to the low prevalence compared with other countries.

The cumulative number of confirmed cases in the general Vietnamese population during the survey period was 7625 (0.008%) and 2,275,727 (2.32%) at the beginning of June 2021 and at the end of January 2022, respectively^16^. Although the prevalence could not be directly compared, our study findings might indicate that there is no clear upward trend in COVID-19 infection among PLHIV compared with the rate of infection among the general population. In the existing literature, the risk of SARS-CoV-2 infection in PLHIV has yielded mixed results thus far. A systematic review and meta-analysis reported that HIV-positive individuals have a significantly higher risk of SARS-CoV-2 infection than HIV-negative individuals^29^. Conversely, other studies have shown that PLHIV have an equivalent risk of COVID-19 compared with the general population^25,30,31^. In Vietnam, PLHIV populations may be cautious about SARS-CoV-2 infection given their health status, and they can easily receive information and advice on preventing SARS-CoV-2 infection during regular hospital visits. Furthermore, measures by the Vietnamese government to protect PLHIV from SARS-CoV-2 infection, such as ensuring priority access to vaccines^32^, might have contributed to preventing SARS-CoV-2 infections among PLHIV.

The tendencies to reduce risky health behaviors (i.e., alcohol consumption, illicit drug use, and risky sexual behaviors) were also consistent with findings from the first survey in 2020 and from those in other countries^33–36^. However, a study from China observed a rebound in HIV risk behaviors among men who have sex with men after lockdown measures were lifted^37^. Furthermore, a study from Vietnam found worrying signs that stay-at-home orders and financial challenges regarding buying drugs could facilitate unsafe injection, group methamphetamine use, and unsafe sex among people who use drugs^38^. Moreover, whereas a decrease in alcohol consumption was reported, binge drinking was associated with depression in this study. Alcohol consumption is culturally perceived as a masculine trait in Vietnam, and there is high social pressure to drink heavily^39^. In our previous work, we found that substance use, including drinking alcohol, was a stress-coping strategy in the PLHIV population in Vietnam, and it was strongly associated with depression^40^. In particular, as the COVID-19 pandemic continues, limited social contact may lead to feelings of loneliness^41–43^, which can be exacerbated in PLHIV who often already have such negative feelings^44,45^. The prolonged COVID-19 pandemic could accelerate alcohol consumption as a mean to cope with emotional stress and chronic uncertainty^46,47^. Therefore, continuous monitoring of risky health behaviors is important.

The economic impact of COVID-19 has remained enormous in the PLHIV population. Overall, the unemployment rate of 17.8% (26.9% in Hanoi vs. 9.5% in other provinces) found in the present survey was far higher than national data in 2021 (4.3% in urban areas vs. 2.5% in rural areas)^48^. Additionally, more than 60% of study participants reported experiencing financial difficulty whereas only a few had received public financial assistance. The Vietnam government issued Resolution No. 68 to support employees and employers facing difficulties owing to the COVID-19 pandemic (effective on July 1, 2021), totaling 1.14 billion USD. However, owing to the complexity and length of the application process, many gave up seeking aid^49^. Given the difficulties for PLHIV to find employment in general, there is a need to accelerate intensive employment assistance as well as capacity development such that these individuals can earn a stable income, which will help to minimize the negative impact of COVID-19 and future crises in this population. Additionally, the government of Vietnam should create financial assistance mechanisms to support those most in need in a timely and efficient manner during times of emergency. One example would be a system like that in the United States, which allows people in need to learn what types of financial aid they are eligible for through websites and apps and allows them to apply online^50^.

Consistent with other studies conducted among the general population in Vietnam^51,52^, a change in employment status and poor financial status were strongly associated with depression. However, the prevalence of depression decreased by more than 10% in our NHTD cohort, in comparison with that of the pre-COVID survey in 2016. This is contrary to the available literature showing an increased risk of mental health problems globally during the COVID-19 pandemic^53^ as well as in Vietnam^54^. There is no apparent reason for this decline in prevalence. However, nearly all NHTD patients (98.1%) reported receiving social support to continue ART and HIV care in the 2021 survey. Additionally, our previous studies found that social support is a strong protective factor against depression in Vietnamese PLHIV^15,19^. Therefore, the reduced prevalence of depression in the present study may be partly explained by enhanced social support during the fourth COVID-19 outbreak. The variable social support was not statistically associated with depression in this study; however, our finding could be due to the fact that nearly all patients in the NHTD received social support. We found significant hospital-to-hospital variation in the proportion of patients who reported receiving social support to continue HIV treatment during the pandemic. Further investigation is needed regarding the reasons for this variation and specific social support characteristics that could protect against depression.

This study has several limitations. First, we adopted a cross-sectional design; therefore, we were unable to determine any causal relationships. Second, COVID-19 outbreaks and the responses varied widely by region and by survey timing, which may limit the generalization of our findings to other regions and pandemic phases. Additionally, the 11 ART facilities that participated in this study were selected in consultation with Vietnam's Ministry of Health from multiple perspectives, including region and facility level. However, the representativeness of PLHIV participating in this study across Northern Vietnam has not been fully validated. Third, the impacts of COVID-19 may be greater for those who were unable to participate in the survey than for those who participated. Continuous follow-up and monitoring would be required for those patients. Fourth, mental health was assessed only among participants from the NHTD, a national-level facility in Hanoi. To further investigate the protective role of social support against depression, future research should expand the number of study sites and include various social support settings. Additionally, more rigorous evaluation of social support characteristics is required. Finally, whereas our study provides important information on the impacts of COVID-19 among PLHIV from the patient’s perspectives, the engagement of families, health care professionals, communities, local governments, and policy makers is essential to make decisions about effective interventions to reduce adverse effects of COVID-19. A variety of research methods, including face-to-face interviews with stakeholders, can help to identify the strategies that are particularly relevant to PLHIV living in Vietnam.

In conclusion, the overall prevalence of SARS-CoV-2 antibodies was low at 1.2%; there was no clear upward trend in COVID-19 infection among PLHIV compared with the rate of infection among the general population. Amidst the stringent social restrictions imposed during the largest outbreak of COVID-19 in Vietnam, HIV treatment was generally maintained and no increase in risky health behaviors was observed. The economic impacts on PLHIV remained significant, with a high unemployment rate, poorer economic security, and binge drinking strongly associated with depression. However, the prevalence of depression was decreased compared with that during the pre-COVID-19 period. Social support, including for patients to continue HIV treatment and effective employment/financial assistance, may help to alleviate the negative socioeconomic impacts of the COVID-19 pandemic and improve mental health among PLHIV.

### Supplementary Information

Supplementary Tables.

## Data Availability

The datasets generated during and/or analyzed during the current study are available from the corresponding author on reasonable request.
