# Supplementary material for: Multicenter observational survey on psychosocial and behavioral impacts of COVID-19 in people living with HIV in Northern Vietnam
Source: Sci Rep. 2023 Nov 21;13:20321. doi: 10.1038/s41598-023-47577-9 (PMC10663486; doi:10.1038/s41598-023-47577-9)
Supplement: Supplementary file 1 — Supplementary Tables. [file 41598_2023_47577_MOESM1_ESM.docx]

Supplementary Table S1. Support to continue ART and HIV care during the fourth COVID-19 outbreak by facility

| Hospital | Received support n (%) |
| --- | --- |
| HDHTD (Hai Duong) | 927 (100.0) |
| 09 hospital (Hanoi) | 401 (98.8) |
| NHTD (Hanoi) | 1,095 (98.1) |
| NTL (Hanoi) | 1,486 (98.1) |
| HTCDC (Ha Tinh) | 338 (97.1) |
| YBMC (Yen Bai) | 94 (92.2) |
| DDGH (Hanoi) | 733 (90.6) |
| TSMC (Phu Tho) | 68 (49.3) |
| NAGH (Nghe An) | 251 (38.2) |
| QNGH (Quang Ninh) | 218 (18.3) |
| HYTD (Hung Yen) | 107 (17.8) |

NHTD: National Hospital for Tropical Diseases, QNGH: Quang Ninh General Hospital, HDHTD: Hai Duong Hospital for Tropical Diseases, DDGH: Dong Da General Hospital, NAGH: Nghe An General Hospital, HYTD: Hung Yen Hospital of Tropical Diseases, 09 hospital: 09 hospital, HTCDC: Ha Tinh Center for Disease Control and Prevention, NTL: Nam Tu Liem Health Center, TSMC: Thanh Son District Medical Center, YBMC: Yen Binh District Medical Center, ART: antiretroviral therapy.

Supplementary Table S2. Needs regarding effective social support to continue HIV treatment

| Free comments in text | n (%) |
| --- | --- |
| **All** | 3,484 (100.0) |
| **Continuous provision of ART and HIV services** | 2,157 (61.9) |
| **Special arrangements to continue ART** | 402 (11.5) |
| Multi-month prescription | 316 (9.1) |
| Drug delivery via post or shipping | 41 (1.2) |
| Provision of medicine at convenient locations | 19 (0.5) |
| Allowing family or others to receive ART from the hospital | 10 (0.3) |
| Favorable environment for hospital visits to avoid contracting COVID-19 | 5 (0.1) |
| Other | 14 (0.4) |
| **Information on COVID-19 and HIV services** | 395 (11.3) |
| **Financial or material support** | 363 (10.4) |
| Financial support | 229 (6.6) |
| Provision of material and/or food | 94 (2.7) |
| Free testing and/or medicine | 22 (0.6) |
| Support with transportation | 18 (0.5) |
| Support with health insurance (e.g., exemption from insurance premiums) | 5 (0.1) |
| **Emotional and mental support** | 179 (5.1) |
| **Support for COVID-19 prevention (vaccines, other)** | 20 (0.6) |
| **Other** | 103 (3.0) |

^a^ A total of 3,484 comments entered.

ART: antiretroviral therapy.

Supplementary Table S3. Comparison of economic impacts between Hanoi and other provinces

|  | Hospitals in Hanoi n (%) | Hospitals in other provinces n (%) | *P* **^a^** |
| --- | --- | --- | --- |
| All | 3,846 (100.0) | 3,962 (100.0) |  |
| **Change in employment status** |  |  |  |
| No change | 1,536 (40.5) | 2,598 (65.6) | <0.001 |
| Lost job | 1,018 (26.9) | 375 (9.5) |  |
| Reduced working hours | 1,141 (30.1) | 879 (22.2) |  |
| Increased working hours | 34 (0.9) | 21 (0.5) |  |
| Other ^b^ | 62 (1.6) | 88 (2.2) |  |
| **Current financial status** |  |  |  |
| No problems | 1,281 (33.4) | 1,376 (34.7) | 0.29 |
| A little challenging | 1,655 (43.2) | 1,718 (43.4) |  |
| Very challenging | 893 (23.3) | 867 (21.9) |  |
| Other | 3 (0.1) | 1 (0.0) |  |
| **Financial assistance from public authorities** |  |  |  |
| Ever received | 192 (5.0) | 199 (5.0) | 0.99 |

^a^ Chi-square test.

^b^ Including those who newly started work, changed jobs, and other.
